# The Super Spreadsheet: collaborative information infrastructure in translational teams

**DOI:** 10.3389/fpsyg.2025.1724977

**Published:** 2026-01-12

**Authors:** Shruthi Venkatesh, Betsy Rolland

**Affiliations:** Michigan Institute for Clinical and Health Research, University of Michigan, Ann Arbor, MI, United States

**Keywords:** team science, translational teams, information management, collaboration, collaboration infrastructure

## Abstract

**Introduction:**

Science teams rely on multiple types of information to communicate, coordinate, and collaborate on their research and operational endeavors. While the Science of Team Science (SciTS) offers robust literature on how such teams use and manage their data, we know relatively little about their collective information behaviors. This paper, the first in a series, reports findings from the Information Management Prototype for Clinical and Translational Research (IMPACT-CTR) study, which examines collaborative information infrastructure within one type of science team: clinical and translational research teams (CTRTs).

**Methods:**

We interviewed 48 team members across 10 U.S.-based teams.

**Results:**

Analyses revealed that CTRTs progress through the research lifecycle of their collaborations by maneuvering several types of information dispersed across various tools, while simultaneously managing their individual information organizational styles in relation to those of their team members. Unlike data, the role of information in their work was often overlooked or undervalued. Teams with misaligned information management experienced time lags and slower project progress, whereas alignment fostered fluency in their work.

**Discussion:**

By generating a typology of information and identifying tensions between individual and collective information practices, this study facilitates our understanding of how to support rigorous and reproducible team science practices in CTRTs.

## 1 Introduction

Roy, a Project Manager at a study site for a larger Consortium project, starts his day by checking *Asana notes* for his to-do list. He then sends a meeting reminder on *Teams chat* for the weekly all-team meeting at noon and *emails* both the participant and the research assistant scheduled for a visit later in the afternoon. During the meeting, he takes notes on the *shared agenda* and assigns tasks to his research coordinators and assistants. To oversee the participant visit, Roy ensures *the Manual of Procedures* is current, prepares the *biospecimen kit*, and pulls participant information from the *visit tracker*. He also keeps the *accounting log* open to record the reimbursement once it occurs. Throughout the day, he monitors the *listserv email* to avoid missing any Consortium-level updates to standardized protocols he would then be responsible for adding to the *regulatory binder*.

Teams run on information. Johnson (2017) posits that a team is primarily formed as an information processing unit, where success depends on how effectively information is gathered from different sources, interpreted, and used in a way that is representative of the efforts of the team members. Science teams are no different, relying on information for both their scientific work and organizational work (i.e., communication, coordination, and collaboration). While the field of the Science of Team Science (SciTS) has learned a great deal about how science teams work with data (Bardyn et al., 2012; Cunha-Oliveira et al., 2024; Ismail et al., 2020; McDavid et al., 2021), we know little about the specific types of information being managed by individual collaborative scientists and even less about the information needs of science teams as a collective.

Information, the human-generated digital objects required to produce data (Chladek et al., 2023) is ubiquitous. As knowledge workers, scientists are inundated with and overloaded by information and tools to manage that information as they work to achieve their scientific and professional goals (Karr-Wisniewski and Lu, 2010). In this vignette, we see Roy, a Project Manager in our study, juggling ten types of information and four different tools to complete the work needed to fulfill his role on the team. We see how he pieced together information to not only accomplish his own tasks but also to oversee those of his team. Roy is not unique in either the complexity of the information required to complete his tasks, or the constellation of tools he uses to do so.

In the realm of team science, specifically, the information needed to achieve scientific goals across labs, across disciplines, and across institutions is complex and often overlooked. A recent commentary on the past forty years of progress in team science finds that high team functionality and efficiency are achieved through practices such as task interdependence, psychological safety, communication, and shared mental models (Salas et al., 2025). Information management is a critical element of such communication and coordination in teamwork. Previous research has shown that this lack of attention by science teams to their information management strategies creates challenges for teams in developing the strong team processes and team dynamics that lead to improved scientific outcomes and outputs (Kelly et al., 2023). Reportedly, knowledge workers spend about 20% of their time searching for the information they need to complete tasks (McKinsey Global Institute, 2012) underscoring the inefficiencies that arise due to poor information practices.

Further, the management of information is a *sociotechnical* problem, one that involves both the complex relations of people working together and the technical aspects of computer-based systems (Baxter and Sommerville, 2011). The collaboration that arises from the coordination of tools, members, and tasks requires teams to understand what information they need, the sources and meaning of such information, and how that information will be managed and shared (Arrow et al., 2000). Indeed, individual teams are complex systems unto themselves (Arrow et al., 2000).

The work described here focuses on understanding collaborative information infrastructure within one type of team: clinical and translational research teams. Through interviews with team members from across the United States, we identify the people, tools, and resources these teams require to conduct networked science.

### 1.1 Information in clinical and translational research teams

The conduct of CTR requires a team-based approach; as such, it is critical to a team's success that information is transmitted accurately across members, tasks, and stages of these scientific endeavors. Clinical and translational research teams (CTRTs) are typically composed of interdisciplinary professionals who aim to integrate basic, patient-oriented, and population-based research to translate scientific findings into practical health outcomes for targeted populations (Rubio et al., 2010; Woolf, 2008). These teams comprise at least 12 different roles such as a basic scientist, clinical research coordinator, data analyst, and patient navigator (Gonzales et al., 2020), and rely on competencies like team communication, research management, and collaborative problem solving (Lotrecchiano et al., 2021) to ensure projects move along the translational continuum. The diversity of roles, along with the varied disciplines and professional backgrounds of translational team members, necessitates strong coordination systems to alleviate time and resource burdens on the team. Delineating the research activities of CTRTs, from the creation of the research question to disseminating the findings, rather than focusing only on the product of the scientific work, can strengthen reproducible research practices (Rolland et al., 2021a). Such networked science entails collaborative, operational, data-related, and structural work aimed at achieving the team's scientific objectives through high-quality data collection and effective organizational and collaborative processes (Rolland et al., 2017). Thus, it is crucial that information flows seamlessly for CTRTs to achieve collaborative success.

Previous research has shown that information management and behaviors such as how team members store, retrieve, share, and use information are foundational to the work of CTRTs (Chladek et al., 2023). Principal investigators of six biomedical teams shared how the ineffective management of physical and electronic information, along with mismatched file labeling, created bottlenecks with collaborators and narrowed the scope of scientific publications (Myneni and Patel, 2010). Similarly, a pilot study with ten members of CTRTs at the University of Wisconsin–Madison offers insights to their information management practices. Interviews revealed that translational researchers and staff often fail to recognize the centrality of information in their work, have conflicting individual information management practices, and receive little institutional support in navigating the different tools to manage information (Chladek et al., 2023). Inconsistent and piecemeal approaches compounded by inter-institutional barriers to information sharing hindered the development of strong team processes; whereas, a culture of trust was created for a few participants whose teams valued transparency and accountability in their information practices (Kelly et al., 2023). However, while this pilot sample included individuals with different roles on their teams (i.e., professor, scientist, research specialist, and postdoctoral researcher), the small sample size and cross-team representation did not allow for exploration of information needs by role. Given the uniqueness of the composition of translational teams and the variety of roles they typically encompass (Gonzales et al., 2020), we are yet to uncover whether, and to what extent, information needs differ by roles within CTRTs. For instance, information barriers may be experienced differently by new team members, by external collaborators brought in for specific expertise, or by lead PIs who maintain a high-level view of the project.

Thus, there is a dearth of literature on the *collective* information behaviors of CTRTs who rely on each other's expertise to create the infrastructure and processes of their research. While extant literature has identified team competencies, institutional processes, and team development interventions that enhance team science in CTRTs, and have begun to examine information management challenges of individuals in CTRTs (Begerowski et al., 2021; Brasier et al., 2023; Chladek et al., 2023; Hall et al., 2018; Kelly et al., 2023), this gap presents a barrier to advancing collaboration in CTRTs, and by extension, effective team science practices.

### 1.2 Data vs. information management

The National Institute of Health (NIH) defines “scientific data” as that which is needed to validate and reproduce findings, but does not include materials like laboratory notebooks, communications, and manuscript drafts (NIH, 2023). Whereas, these materials are classified as “information” or the human-generated digital objects required to produce such data (Chladek et al., 2023). However, the knowledge base of what constitutes information for CTRTs is limited.

Given the NIH's emphasis on sharing and replicating scientific data, and the notion that quality data is an indication of scientific output and progress (Myneni and Patel, 2010), previous research in data management has extensively categorized best practices, the types, tools, repositories, and methods for researchers to manage and store data (Borghi et al., 2018; Cunha-Oliveira et al., 2024; Ismail et al., 2020; Luo et al., 2016). For instance, a review was conducted on the various decentralized clinical data management technologies used in U.S. academic research centers (Cummins et al., 2023), another examined the data support services provided by universities (MacDougall and Ruediger, 2024), and others explored researcher challenges and benefits that stemmed from availing of such services (McCracken and MacDougall, 2025; Wiley and Burnette, 2019).

Corresponding bodies of knowledge for information management in scientific research, and, by extension, clinical and translational science, are lacking. For collaborative scientific teams, data work is only one component of the broader activities that they engage in (Rolland et al., 2017). Just as the biomedical and clinical research fields have expended considerable effort to evaluate data management, we must also understand the types of information that are required to coordinate across disciplines and domains of expertise in CTRTs. Such an understanding will strengthen our ability to support rigorous and reproducible team science practices in translational teams (Rolland et al., 2021a).

### 1.3 The current study

We report on Information Management Prototype for Clinical and Translational Research (IMPACT-CTR), a National Library of Medicine-funded study investigating the information behaviors of CTRTs. Drawing from Chladek et al. (2023), we define information as human-generated digital objects that are manipulated to produce data and carry out scientific work through behaviors such as creating, seeking, storing, retrieving, or using. The study is the first of its kind, to our knowledge, to examine the information management practices of CTRTs across the U.S. By focusing on CTRTs as one specific type of science team, through a series of manuscripts, we aim to propose a conceptual framework for evidence-based information management practices that impact team functionality.

In this paper, we extend and build on our pilot studies (Chladek et al., 2023; Kelly et al., 2023) by including a larger sample of CTRTs across more teams and roles. This and future papers on the study will follow the conceptual model, the Translational Team Science Hierarchy of Needs, which was inspired by Maslow's hierarchy of needs (Kelly et al., 2023). Herein, the authors purport that for CTRTs to achieve the peak of “translational nirvana,” their basic needs of collaborative infrastructure and information management processes must first be met. Meeting these basic needs, in turn, could influence strong team processes and psychological safety that would conceivably enable them to reach the apex of research synergy where infrastructure, expertise, clear communication, and shared mental models converge to spur the team's effective achievement of their scientific goals.

In this manuscript, we establish the evidence base for the first rung of the pyramid: Collaborative Infrastructure, which refers to the people, tools, and resources that CTRTs require to carry out their work. In order to understand the barriers and facilitators of information management in CTRTs, we must first establish a typology of information these teams engage with. To this end, our research questions are:

RQ1: What does “information” for CTRTs comprise of? How are tools used to manage such information?

RQ2: How do CTRT members navigate their team's information systems?

## 2 Materials and methods

The study was marketed via a broad recruitment campaign, including outreach to Clinical and Translational Science Award (CTSA) Hub admins and current K and pilot awardees (see Venkatesh and Rolland, 2025; for details of the recruitment process). Our eligibility criteria were funded U.S.-based CTRTs with at least four members, including the principal investigator (PI). Teams could be at any stage of the translational process. As of this writing, 10 CTRTs have virtually participated in our study.

Once a team member expressed interest in the study, we scheduled an informational call with the PI to discuss study participation and steps. After the team confirmed participation, brief enrollment surveys were distributed to team members via REDCap. These included demographic information and questionnaires about their teamwork processes and psychological safety used in prior work (Edmondson, 1999; Rolland et al., 2025). Then, they participated in a one-hour individual interview on Zoom with the first or senior author about the team's processes for seeking, sharing, storing, and using information. The semi-structured interview protocol included questions about how decisions were made and documented during team meetings, the collaborative writing process the team follows, any guidelines or protocols that existed for lab norms and operations, and the alignment between individual and team information management practices (see Appendix). The average interview length was 46 min.

A subset of teams (*n* = 4) was asked to allow a study team member to observe their team meeting to complement insights from the individual interviews. Participants who described a unique use of a software or tool were also requested to engage in a brief user-experience interview about that tool (*n* = 6). To round out the study (and as a thank-you for participation), the teams were offered a 90-min facilitated Collaboration Planning session. Collaboration Planning is an evidence-based intervention developed by the senior author that enables teams to think more intentionally about how they collaborate (Rolland et al., 2021b, 2025). Seven teams participated in the session. To conclude study participation, participants completed a brief survey after the Collaboration Planning session about team processes and feedback on the session. This study was deemed exempt by the University of Michigan's Institutional Review Board, HUM00255826.

### 2.1 Participants

Our analytic sample included 10 CTRTs with a total of 48 participants. Team size ranged from three to seven members (including the PI), with an average of five members per team. Thirty participants (63%) identified as White, 43 (90%) as Not Hispanic or Latino, and 29 (60%) were female (Table 1). In terms of geographic distribution across the U.S., four teams were primarily based in the South, three in the Northeast, two in the Midwest, and one in the West. Notably, two teams included individual members or collaborators located in a different region from the primary PI. Although our recruitment criteria requested a minimum of four members per team, for two teams, we ultimately got only three participants each. We have included these teams, recognizing that their insights remain valuable and that they had distinct roles within their teams.

**Table 1:** Participant demographics.

**Demographic category**	**# (%)**
**Primary role**	
Faculty member	19 (40%)
Contact PI	*10*
Staff	16 (33%)
Postdoctoral scholar	*3*
Research specialist	*3*
Research coordinator	*10*
Project manager	7 (15%)
Research assistant	6 (13%)
**Geographic distribution**	
South	4
Northeast	3
Midwest	2
West	1
**Race and ethnicity**	
Not Hispanic or Latino origin	43 (90%)
Hispanic or Latino origin	5 (10%)
White	30 (63%)
Asian	10 (21%)
Black or African-American	3 (6%)
Native Hawaiian or Other Pacific Islander	1 (2%)
More than one race	2 (4%)
Other	2 (4%)
**Gender**	
Female	29 (60%)
Male	18 (38%)
Non-binary/gender non-conforming	1 (2%)

With respect to the team roles that were represented, we had 19 Faculty Members including our 10 contact PIs; 16 Staff members including Postdoctoral Scholars (*n* = 3), Research Coordinators (*n* = 10) and Research Specialists (*n* = 3). Additionally, we had 7 Project Managers and 6 Research Assistants (see Appendix for how these roles were categorized). Each team had at least three members of different roles/designations. Of the 38 participants who shared their team tenure, 5 of them (13%) had been with their teams for less than a year, 23 of them (61%) for 1–4 years, and 10 of them (26%) for more than 4 years.

As identified by the PIs, five teams focused primarily on behavioral and social science, two on biomedical science, two on engineering, and one on clinical science. Four were working on funded projects that included collaborators from more than one institution, while three involved collaborators from different departments or laboratories within their home institution. Two teams served as sites for larger consortium projects, one team focused on a lab-specific project, and one team provided research coordinator support through their university's CTSA.

### 2.2 Data analyses

Audio and video recordings of the Zoom interviews were deidentified using pseudonyms and stored securely on Dropbox. Transcripts were generated from the audio recordings by a third-party service, quality checked by a study team member, and the video recordings were subsequently deleted.

The coding process began with a hybrid approach that combined inductive and deductive methods (Fereday and Muir-Cochrane, 2006), using NVivo. We first indexed codes according to the questions in the semi-structured interview protocol (Deterding and Waters, 2021). A codebook was then developed by analyzing 10% of the sample transcripts, with one randomly selected transcript from each of the first five teams to ensure variety in representation. Inter-coder reliability was established through discussion between the first author and a research assistant by coding 15% of the transcripts (Kappa = 0.72). We followed an iterative process to refine the codebook and resolve any disagreements. Following this, the first author independently coded the remaining transcripts for the purposes of this paper.

Codes were grouped and queries were generated to identify emerging themes. It is important to note that this manuscript focuses only on a subset of codes relevant to the research questions elucidated earlier from individual interview data. Findings from other data sources such as surveys, user interviews, meeting observations, and Collaboration Planning, will be reported in subsequent manuscripts.

## 3 Results

Three themes emerged from our data on the collaborative infrastructure (resources, tools, and team members) required by CTRTs: (1) CTRTs engage with innumerable types of information, adding layers of complexity and tacitness to their collaborative research work, (2) CTRTs use multiple tools simultaneously to manage information, with varying levels of training and to varying degrees of efficiency, and (3) CTRT members navigate the “me vs. we” in information management, resulting in struggle, adaptation to team practices, or alignment fluency (Table 2).

**Table 2:** Representative quotes.

**Theme**		**Representative quote**
CTRTs engage with innumerable types of information, adding layers of complexity and tacitness to their collaborative research work	Information is complex	“We got in the habit of having electronic regulatory documents. So we just scan those forms and save them to the regulatory file of the OneDrive. And I also print a PDF of all of our email correspondence, and then save that in OneDrive as well. For a lot of studies, we mine data from their [patient's] electronic medical record. We just search within the encounters or their lab results and then either write it down on a case report form, a paper worksheet, or enter it directly into the electronic data capture system…we also have an all-Teams chat, and we're constantly asking each other questions.” (Fiona, Research Coordinator)
	Tacit information	“She [PI] just gives an overview of the projects. So people can kind of tune in and out of that hour to know, oh, this doesn't pertain to me, and this does pertain to me. So she kind of restates that, I think, at the beginning of every meeting, but that's not really written down anywhere. It's just really how she functions. Obviously, all of her study protocols are written down somewhere. But in terms of how we function as a team, no.” (Iris, Faculty member)
CTRTs use multiple tools simultaneously to manage information, with varying levels of training and to varying degrees of efficiency	Tool functionality	“I think that the studies that I've had the most success with Microsoft Planner are studies where there's kind of lots of tasks that need to get done.” (Finley, Research Coordinator)
		“We upload our [visit] videos to a consortium wide website, and our critiques. But once the data gets pushed, it doesn't take me to a review scores page, it just submits it all, and we actually don't have access to it after that. I struggle with not being able to report scores to [participants] after that assessment is pushed.” (Katherine, Faculty Member)
	Interoperability of multiple tools	“[The transition] has been terrible. It's still in process, honestly. It's even more complex in our institution because we had an internal shared drive, then it switched to OneDrive, most recently to Teams. So right now, we kind of have all three systems' and so different files get saved in different places. And that makes it challenging when we need to find them. So that's the biggest impact is that it takes greater time to identify things.” (Mia, Faulty PI)
	Technology savviness of team members	“Some of the team members, maybe because they're curious, they try to update it [tracker] on their own or try to do different things in it. And this is a shared document, so when someone tries to go in and makes some adjustments or updates things, and they don't do it correctly, then it makes it hard for everyone else to do it.” (Maya, Faculty Member)
CTRT members navigate the “me vs. we” in information management, resulting in struggle, adaptation to team practices, or alignment fluency	Alignment fluency	“I think it's very clean and easy to follow, and it's very basic. There's information that passes from one person to the next, and it's like a baton, right? It's like you're trying to get to the final end of the race.” (Benjamin, Project Manager)
	Aligned, with adaptations	“I think the biggest thing is theirs [the team's folders] just went one layer deeper. For instance, they would break things down for the experiment by the day. So within each experiment, there would be a folder for its own specific day and that would be where that day's raw data and everything is. Whereas, I would just kind of throw everything into that experiment. I never really split it up per day, because I'm like, ‘it's timestamped. Why would I really need to do that?' But…it just makes things cleaner at this point… it kind of creates a sense of transparency, because then you can see what everyone's doing.” (Caleb, Research Assistant)
	Misaligned	“Oh, we're probably different. I honestly think that once I stop doing it, it won't happen. I don't think that others are really aware of how much information we have that needs to be organized. So I would say different in that sense.” (Kayla, Research Coordinator)

### 3.1 Theme 1: CTRTs engage with innumerable types of information, adding layers of complexity and tacitness to their collaborative research work

“We had a meeting, and we started to realize that we need some information about results and protocols. So I was instrumental in creating folder numbers. So we had experimental logs, and in the experimental logs, we have folder that is related to which team, and we have a master log…then we have lab notes…we are good at updating some of the stuff but not all the stuff.” (Nancy, Research Specialist)

**Figure 1:**
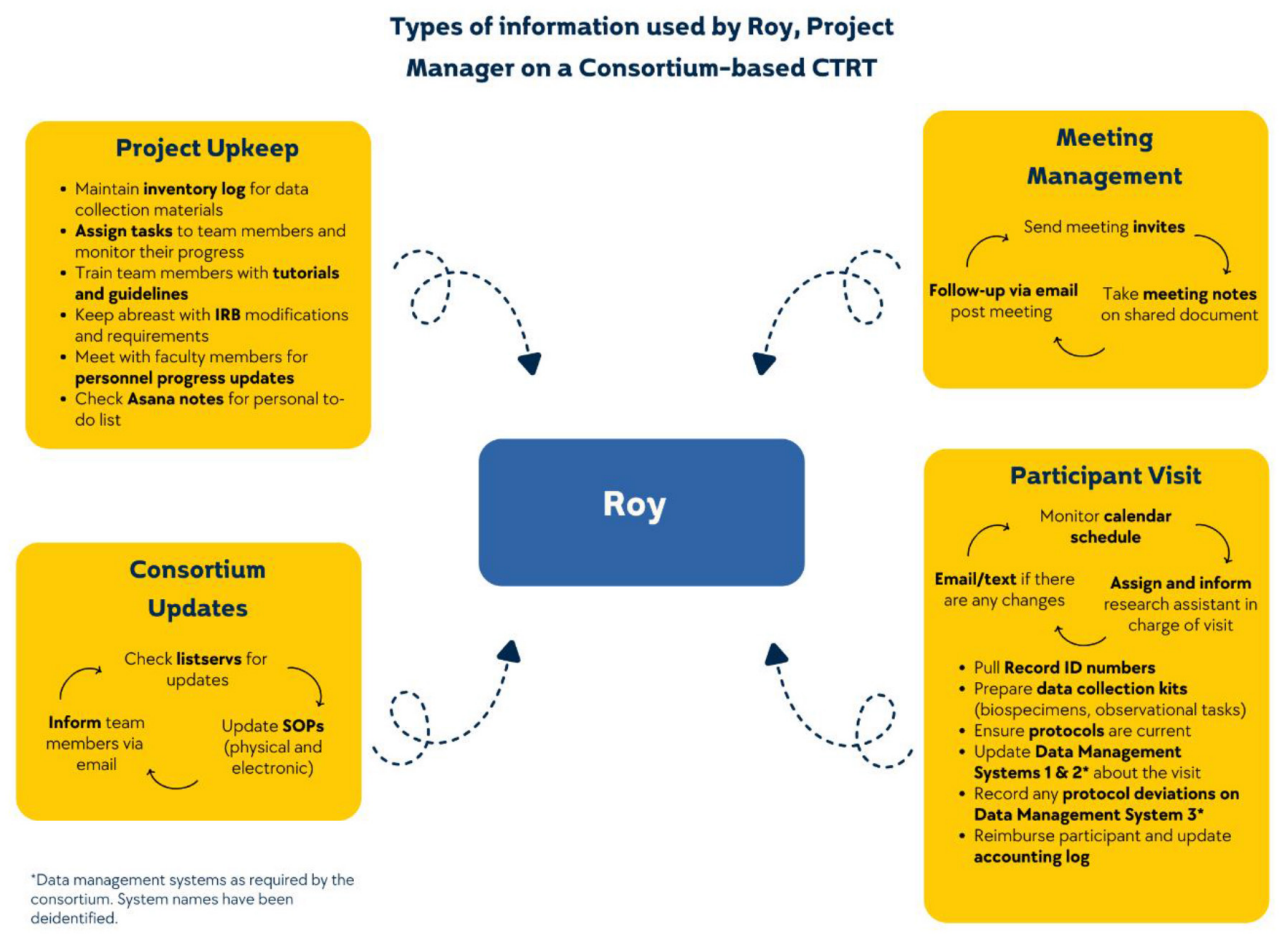
Illustrative example of information types used by a CTRT member.

CTRT members engaged with innumerable types of information to carry out their collaborative research goals. As exemplified in the initial vignette (Figure 1), Roy required many pieces of information even to complete one task of managing meetings. First, he needed to know team members' availability and send calendar invites. Then, on a recurring basis, he had to prepare a meeting agenda, take notes during the meeting, and follow-up with task assignments post the meeting. Similarly, Nancy, who was part of a team that collaborated across four labs, shared how she managed experimental logs for each lab's team, maintained an overall log to track each lab's experiments, and consulted lab notes to supplement the trackers. This way, information was numerous in terms of the type and quantity, adding complexity to already challenging work.

Consequently, while information around data collection, tracking, and analyses tended to be heavily protocolized, the processes by which team members carried out their roles largely remained vague, tacit, and “in their heads,”

“There's not a protocol for doing the work. There's a protocol for the study, and then you know what you have to do to make that happen. Everybody on our team has been here for a few years, and I think it's really just kind of a learn as you go. There's not much of a plan in place ahead of time.” (Daphne, Research Coordinator)

We saw substantial tacit knowledge around *how* to complete many tasks. This lack of documentation or discussion about how the work actually got done meant that team members did not always recognize the importance of information in shaping their work. To illustrate, Daphne knew her team had an established protocol, regulatory binder, REDCap surveys, biospecimen kits, and a timekeeping log to follow during a participant visit. But the steps of how to use these different materials to collect data was not always linear or discussed, and figuring how to proceed was the “learn as you go” process she described. Moreover, for some team members who experienced such information gaps, this confusion was “time consuming” (Grace, Faculty PI) to work through and detracted from their scientific tasks.

**Figure 2:**
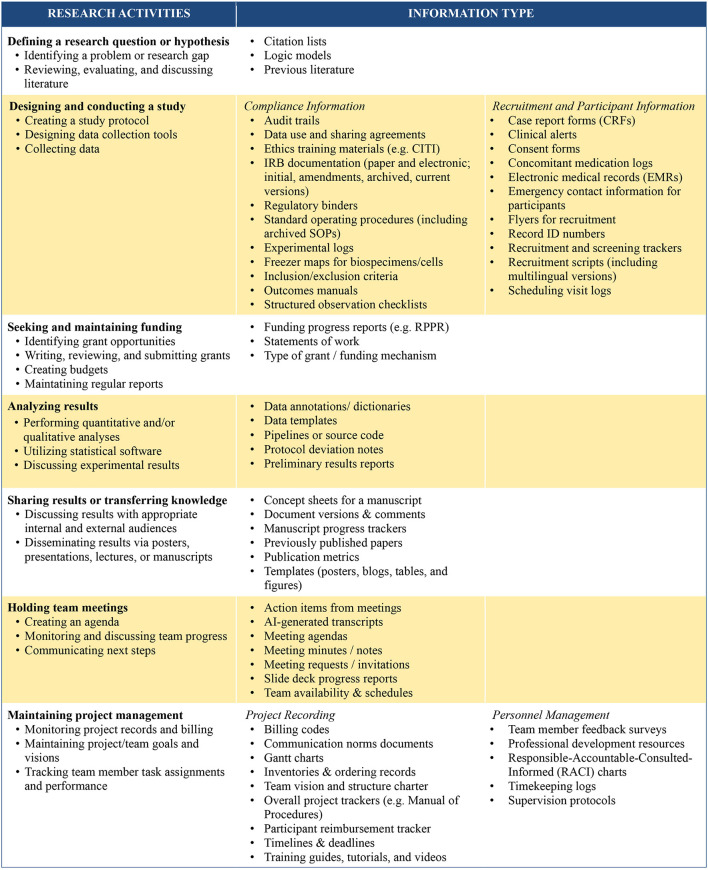
Information types required by CTRTs for different research activities.

Throughout our conversations with participants, a myriad of types of information was described. Here, we present a typology of information for CTRTs, in order to make this tacit knowledge explicit (Figure 2). These types of information are categorized according to the stages of research activities that CTRTs typically engage in, inspired by Figure 3 in Chladek et al. (2023). While these are roughly the stages of a research project, the process is not necessarily linear. It is conceivable that individual team members work on different stages concurrently, such that the team as a whole might require access to most types of information at any given time (e.g., a research assistant could be working on a manuscript while a postdoctoral scholar could be drafting a grant submission). While not an exhaustive list, this typology captures the multitude of information types that can quickly become complex to navigate. Transcripts were coded every time a participant mentioned a distinct type of information that they seek, use, retrieve, or store to carry out the tasks of their roles.

**Stage 1: defining a research question or hypothesis:**

CTRTs required access to previous publications and reference lists as they identified a research gap to explore. Our teams shared little about this stage, as all were already executing their funded projects.

**Stage 2: designing and conducting a study:**

The information related to designing a study and collecting data was broadly classified into two sub-categories: (1) Compliance information and (2) Recruitment and participant information. First, compliance information pertained to procedural requirements, usually set by the funding agency, ethical boards, or institutional policies. It included mandatory documentation and guidelines such as the IRB, standard operating procedures, regulatory binders, data sharing agreements, “For things that are less project related and more globally important to the lab, like a consent form, thankfully, 99% of that is uploaded and stored within the IRB that we have” (Derek, Research Specialist). Second, recruitment and participant information included information about the participants, such as their record IDs, emergency contact information, electronic medical records, visit logs, and case report forms. We note here that these materials are not considered data by NIH (2023); however, such information provided details about the participants that were necessary to subsequently collect aspects like survey responses, biospecimens, or observational behaviors which constitute the actual data of the projects. Furthermore, recruitment information also entailed the resources required for participant and community outreach, including recruitment tracking, recruitment scripts, marketing materials and flyers, “So we have an internal tracker for our recruitment goals and also completing the follow-up calls, so that tracks each month, how many people are recruited into the study and then how many follow-up calls are completed” (Josie, Research Coordinator).

**Stage 3: seeking and maintaining funding:**

As CTRTs maintained their funding and curated applications for additional funding, they used materials such as budgets, statements of work, and progress reports,

“Every year, we have to turn in a progress report outlining our major accomplishments and our plans for next year, as well as a copy of that statement of work, showing which tasks are completed, which tasks are still yet to be completed.” (Bailey, Faculty PI)

**Stage 4: analyzing results:**

To perform data analyses, CTRTs required information about the data, such as protocol deviations, data templates, and source codes to smoothly perform analyses, “So when [we] start to analyze data, we already have our own protocol, we have set up the pipelines. The student just needs our new pipeline to run the data and save the process data to a new folder” (Zachary, Postdoctoral Scholar). To finalize analyses, they went through rounds of preliminary analyses and sharing raw data with each other which generated suggestions and feedback, as Caleb, a Research Assistant noted,

“The goal of the subgroups is that you bring your most recent research with whatever experiments you've been doing in the past two weeks and present on that.. very raw figures and very raw data. The idea is to get input on better ways to design the figures or better ways to analyze the data.”

**Stage 5: Sharing results or transferring knowledge:**

During dissemination preparation, teams depended on information such as previously published material, citation lists, and writing templates. Additionally, some teams who had reached manuscript preparation stage shared their collaborative writing process and how they relied on document versions and tagged comments to keep abreast with their writing assignments, “The [PI] and a few other team members did the bulk of the draft and then kind of tagged a few of us in certain places for us to have focused input and writing” (Cora, Faculty Member). Interestingly, most of our teams did not have written authorship guidelines, though none of our participants reported a conflict around authorship roles. Some reflected they had a shared (unwritten) understanding of authorship order.

**Stage 6: holding team meetings:**

Most commonly, collaborative information sharing occurred at meetings. Almost all our teams met weekly, closely followed by bi-weekly meetings. One team had monthly meetings, and a couple had quarterly group meetings. Majority of our teams had designated note takers for minutes and follow-up. These included the Project Managers, some self-appointed faculty members, and student volunteers. Information for a meeting included meeting requests, agendas, progress reports, and AI-generated transcripts, “I actually record audio of the meeting. Then, I just create an outline using our ChatGPT version, using the transcript from the audio recording...then I share it with [PI], and usually forward it to the team before the next meeting” (George, Project Manager).

**Stage 7: maintaining project management:**

This research activity was added to the original Figure 3 in Chladek et al. (2023) to encompass the operational and administrative information CTRTs used to keep their projects progressing and ensure goals are on track such as Gantt charts, team vision and structure, and logic models. Here, it is interesting to note that a few teams used variations of an overall tracker, or a “Super Spreadsheet.” This served as a centralized tracker of the team's projects, broken down by specific tasks with relevant links, upcoming deadlines, and the names of team members responsible for those tasks. It was designed as a living, editable document that adapted to the team's needs. For those teams who had such a Super Spreadsheet, it became a go-to resource,

“And for the sheet that I told you about, with all the projects, it's just available for everyone to view because it also helps keep track of where everything is. Sometimes if people forget what the deadline is or what the goal is, they go back and refer to it, especially because a lot of the projects have submission deadlines to certain journals and all of that. So it's important to keep all of that in one place.” (Bonnie, Research Assistant)

Project management additionally contained personnel management information, such as supervision protocols and timekeeping logs.

In the background of these activities, all team members actively relied on general information management tools used by nearly all knowledge workers such as email, chat, text, and calendar schedules to move research forward, while several also kept their own personal notes to stay on track. We also acknowledge that these categories are not mutually exclusive: while we have attempted to distinguish them by research activity, information in one category may also serve purposes in another. For example, an IRB document is primarily required at the “designing and conducting a study” stage but can also be referred to during the “sharing results” stage when a team member writes the Methods section of a manuscript to verify the procedures of the project. Taken together, CTRTs rely on multiple types of information as they cyclically progress through the various stages of translational research projects.

### 3.2 Theme 2: CTRTs use multiple tools simultaneously to manage information, with varying levels of training and to varying degrees of efficiency

“[The consortium-mandated participant management system] is frequently down. There's always a problem. And it is a little bit harder to keep track of recruitment metrics because I don't think it's at a stage where it's functional for a project of this size.” (Izzy, Faculty Member)

**Figure 3:**
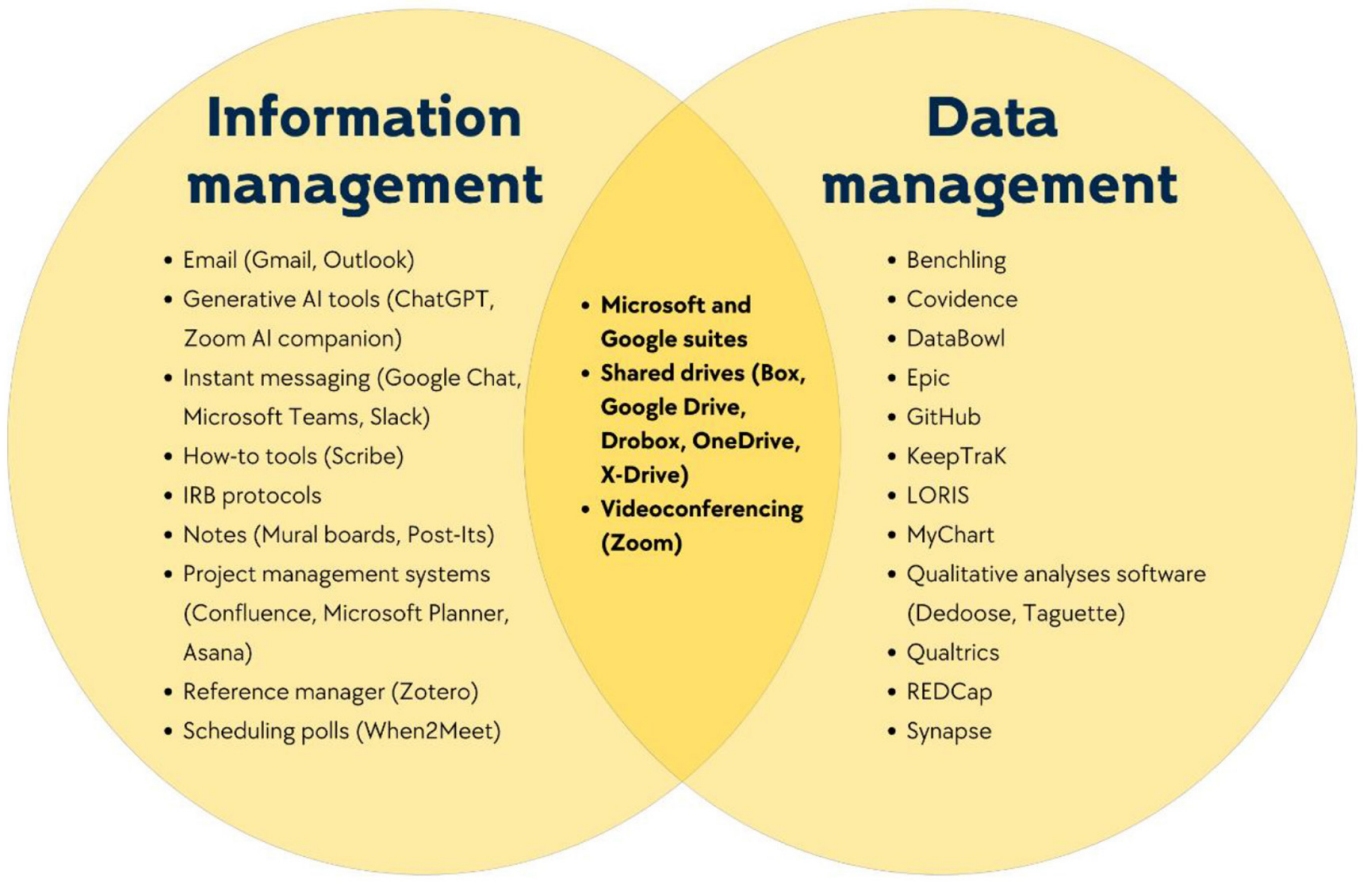
Tools used by CTRTs for information and data management.

CTRTs use, to varying degrees of success, multiple tools that facilitate the information behaviors of seeking, storing, sharing, and retrieving the information types described in Theme 1. Our CTRTs used a range of three to eight tools each: most of these were provided for and required by their home institutions, while others were mandated by funding consortiums. Figure 3 presents the tools mentioned by teams, grouped at the team level. While investigation of data management tools is beyond the scope of this paper, we present them here to provide a complete view of the tools CTRTs engage with, and illuminate the interdependence among data, information, and information about data. On the left side of the figure are information management tools, such as instant messaging platforms (Google chat), scheduling polls (When2Meet), and project management applications (Asana) that assist with communication, task management, and scheduling. On the right side are data management software such as Qualtrics, REDCap, and Dedoose, which teams use to collect, analyze, and maintain their data. At the center of the Venn diagram are intersectional tools, such as Dropbox, which can securely store data while also housing information for CTRTs. For instance, Samuel shared, “We'll store meeting notes on Box. Milestones and goals will be stored there as well. And then in terms of key experimental results and reagents and logs, we'll have storage on there as well” (Samuel, Project Manager).

The efficiency of these tools in hindering or facilitating collaborative research depended on three factors: (1) the functionality of the individual tool, (2) the interoperability of multiple tools, and (3) training and technological proficiency of team members.

Firstly, participants' perceptions of individual tools as either helpful or clunky appeared to depend on the independence they had in choosing them. When team members had agency in selecting a tool for their information management that they subsequently shared with the rest of the team, they had more success with its functionality. For example, Blair, a Research Coordinator had brought her knowledge of Asana, a project management system, to this current study and shared,

“I feel like the utilization of Asana kind of provides a certain equity in the team, where I think of us as colleagues. Between team members, for me, efficiency is key. And why am I going to waste time typing a long email when in actuality, I just need a quick response to keep the project moving?”

Similarly, Bella discovered the usefulness of Scribe in streamlining processes,

“Scribe is an amazing tool to create a step-by-step how-to. So it's very helpful in creating SOPs for protocols. It works by essentially recording your screen. So, you record a process, and it creates a step-by-step PDF for you to share.” (Bella, Research Coordinator)

We did not encounter a situation where participants chose a tool of their own accord that ultimately stalled the team's workflow. Whereas, other members who used certain mandated tools experienced technical glitches that slowed task execution and created information loops, as highlighted above by Izzy. Kayla, a Research Coordinator echoed a similar view about the clunkiness of a platform, “Sometimes it [the platform] will just go down for a day, and then you can't really use it when you need to use it.” Additionally, Josie, a Research Coordinator, explained how their participant tracker was stored in a secure format that only allowed one person to work on it at a time. However, the spreadsheet often showed as “in use” even when no one was accessing it. As the tracker had to be updated before a participant visit, team members found themselves in a loop asking each other who was using it or waiting for the system to refresh, which could sometimes last for hours. This was “frustrating” and even led to visits being rescheduled, hindering progress on the project. Members were inclined to accept these inefficiencies as part of the research process, especially since they had limited ability to change these required tools.

Secondly, CTRT members often used more than one tool simultaneously to accomplish a study task, and in some cases, the interoperability of these tools was lacking. For example, Diana, a Research Assistant, shared how she would access the Manual of Procedures on the shared OneDrive to locate her task assignment, then proceed to REDCap to make the necessary changes to a study survey, and Slack a team member she was coordinating with to update them about the edits. As such, using multiple tools for information and data management was a baseline expectation for team members across roles. In cases like Diana's (and for several team members), there appeared to be a clear distinction between the role each tool played in facilitating their work. However, for some team members, using multiple tools was complicated by the lack of interoperability, resulting in duplication of the work, or time lags between entering information on one system and accessing it in another. Cora, a Faculty Member noted, “We're on multiple different platforms and it's a continual challenge. We just do the best we can.”

Institutional policies on tool use augmented this lack of interoperability. Nearly half of the teams reported having to shift between systems (e.g. from Dropbox to Box) due to institutional changes. Although the changes are implemented at the institutional level, it fell on individual teams to navigate the consequences of the shift in their file storage, similar to Chladek et al. (2023),

“OneDrive has also changed that landscape a lot. For a period of time, we used Box files. Then [institution] severed ties with that, so all that got imported into individual OneDrive folders. And so a lot of those things were super buried and you [would] have to do a lot of investigation to find.” (Charles, Project Manager)

These shifts were often accompanied by file duplication, or files being stored across more than one tool, which made it challenging to find when team members needed them or know which version to use.

Thirdly, despite this inevitability of needing multiple tools, teams differed in the kinds of tutorials available for training members on how to use them. Some relied on screen-share walkthroughs for new or junior members on an as-needed basis, while others had received training guides from the software's website. Several described the process as trial and error, supplemented by extensive “Googling.” This challenge of getting everyone up-to-speed was further compounded by variation in members' technological proficiency, which influenced their ability to effectively use the information systems,

“For some people, creating an Excel file and manipulating it and creating extra sheets, color coding and all this kind of stuff, they know that system pretty well and can do that. And for other people, it's just Greek. If you put it in front of them, they may be able to read it, but they're not going to be able to really utilize that technology in a functional kind of sophisticated way.” (Grace, Faculty PI)

Such differences in skill sets necessitated additional training time from senior members, corrections or re-doing of the work, and in some cases, restoring previous versions of the file on hand, which could create in-the-moment delays in the team's work.

In this way, the work of CTRTs was dispersed across many tools to manage their information, data, and information about data, with teams utilizing these tools to varying degrees of efficiency.

### 3.3 Theme 3: CTRT members navigate the “me vs. we” in information management, resulting in struggle, adaptation to team practices, or alignment fluency

“Our outreach team are less concerned about organization. They like to be able to access things when they need to access them, whereas my research team create folders with a million different subfolders and they're very focused on how things are organized. I'm probably somewhere in between where I like to know where things are, but I don't ever spend time organizing things. One challenge is I have my own set of personal files as well that don't always get uploaded to centralized files. So I'd say there's probably some variation.” (Mia, Faculty PI)

As Mia highlights, team members constantly navigated the “me vs. we” in their information organization i.e., how they reconciled their personal information styles with those of their team's, with little guidance. Specifically, when asked how similar or different participants' own information management was compared to the rest of their team, they tended to reflect on the extent to which their individual file organization aligned with that of their team's. Only two of our teams had written guidelines for what should be stored where within shared drives. As emphasized in Theme 1, it was rare to have protocols on *how* the work is done, in this case, how folders should be structured, “I've never developed a formal taxonomy of file storage for this particular study. I have my own personal file storage, but I haven't copied that over and made it mandatory” (Mabel, Faculty PI). Instead, information structures were verbally communicated or team members “just follow what's in place” (Shane, Faculty PI). This led team members to one of three outcomes; they either: (1) struggle with misalignment, (2) adapt their practices to reach alignment, or (3) experience alignment fluency with minimal adjustments.

Some team members struggled with knowing where to find, store, or locate information they needed to do their work. Navigating file structures within the collective “we” of shared drives was complicated by the fact that each individual “me” had a different intuitive organizational sense, as highlighted by Faith, a Postdoctoral Scholar,

“The files are set up by whoever sets up the files, and files are set up like their brain, and everybody's brains are different. So I have really struggled with finding the right folders for things. I have saved things in what I thought was the right folder, and then somebody says, “I can't find it.” And I'm like, “It's right here.” And they're like, “Why is it there?” That is not intuitive to me, obviously.”

For a few team members, such misalignment impacted the team as they consequently operated off “a different knowledge base than we should” (Lucas, Faculty PI). One participant even noted that when team members fail to recognize the effort taken to organize information, it undermined study progress. There was an acknowledgment by some that though they were probably wasting time searching for things, discussing how to better streamline the system was not a priority, “We haven't talked about it [nomenclature]. It hasn't been a priority. Our priority has mostly been to push ahead and try to get our project going” (Nyla, Faculty Member).

That said, several team members in our sample were in the middle ground of the “me vs. we” like Mia: they recognized that their personal organization differed from their team's, but they adapted for the sake of team functioning. For some, their own information management was less rigid, “So I think it's only because I'm working within an already-established, clearly defined information structure that I follow that, and everything is there. But for whatever reason, it's not what I would naturally do” (Frederick, Research Specialist). Such established shared folder structures with multiple folder trees could be “overwhelming,” and individuals worked through it by either modeling the structure of other member's folders, or deferring to the hierarchy of the team,

“We kind of adopt a top-down direction, so the way that I organize things might not be the way that somebody else would innately organize things. But because I am the one who's more senior in this role, this is the way that it's going to be done.” (Derek, Project Manager)

Whereas, other members preferred more organization and structure compared to what the team currently had in place, and in those situations, they learned to “relinquish control a little bit” (Izzy, Faculty member). Izzy added that this gray area made her engage in “very deep self-reflection around if that's a me problem or a them problem” but had learned to celebrate when members agreed on a workable information structure for the team. In this way, there was a consensus that balancing individual and team-level information structures required members to have a “higher degree of flexibility and [a] problem solving” mindset (Fiona, Research Coordinator).

On the other hand, some members expressed fluency in their information alignment with minimal need for adaptation. The teams appeared to have shared clarity for how information was stored, labeled, and retrieved. While the members described previously attained alignment through compromise of their own information styles, these individuals discussed alignment as one that already existed with no desire for changes as information flowed seamlessly, like a “baton” (Benjamin, Project Manager). This sort of gold standard was encapsulated by Isaac, a Research Assistant,

“We‘re very fluent in the structure that we have. So we are all speaking the same language when it comes to information. We all kind of know exactly what we're talking about on the SharePoint.”

Upon further exploration, this specific team's synchrony appeared to stem from having a clear nested folder structure: an overall folder for the team charter and data sharing templates, followed by individual team member folders, organized by date, for raw and process data such that Isaac knew where his deliverables were to be stored on the shared drive. Even though the requirements of such a structure were not formally documented, frequent communication of these expectations in team meetings was beneficial in establishing a shared understanding. This fluency helped him (and other participants who experienced similar alignment) foster transparency in their team's work.

## 4 Discussion

This study augments our understanding of the crucial role of information in the conduct of clinical and translational research. CTRTs proceed through the research lifecycle of their collaborations by managing multiple types of information spread across various tools, all while balancing their own intuitive organizational styles with those of their team members. Teams with misaligned expectations experienced negative project progress, while teams that were aligned expressed confidence and transparency in their work.

Akin to Chladek et al. (2023), our participants did not always recognize the importance of information in their work, reflecting the complex ways in which information is embedded into conducting research. The typology of information that CTRTs generate, use, and rely on presented here can be used as a guide or rubric for CTRTs to locate where their study stands and to identify what team members may need to feel adequately supported in their work. Importantly, though we chose to organize this typology around the research activities (Chladek et al., 2023) that most research teams navigate, translational research is not linear (Lean et al., 2008), and neither are the research stages of individual CTRTs. As elucidated in Theme 1, within teams themselves, members can be engaged in activities at different research stages. As such, the typology should not be interpreted as a set of requirements, but rather as a framework integrating information systems into team project planning.

Our findings also revealed that team members were more explicitly familiar with data in conducting CTR than with information. Data is often considered the lifeblood of academic research and, given the emphasis on reporting requirements (Zhang and Hughes, 2019), as well as the prominence for scientists being trained to be data literate (Koltay, 2017), members were more likely to be familiar with the guidelines and protocols during the “designing a study and collecting data” and “analyzing data” stage of research activities. By contrast, the multitude of other forms of coordination, such as communications, checklist, meeting notes (Myneni and Patel, 2010) and trackers, were also critical spaces where information about the decisions across the research lifecycle were managed yet undervalued. We argue that information is both complementary to and intersectional with data management in the conduct of translational research. Much of this information was implicit in the everyday doing of the work to the extent some participants felt discussing information and organization was “useless” (Daphne, Research Coordinator) or less important than the “actual work.” However, prior research has ascertained that taking the time to explicitly discuss and plan information practices is itself essential for the team's success in reaching scientific goals (Rolland et al., 2025). It is not that everything must be formally documented to be useful; rather, discussing a shared understanding of expectations can be beneficial, as evidenced by Isaac's description of his team's SharePoint organization.

To this end, unlike previous work where participants were described as “freelance” (Chladek et al., 2023) or “idiosyncratic” (Myneni and Patel, 2010) in their individual information practices, our analysis revealed a general consensus within teams about the tools they used. However, the efficiency of tool use was multi-layered: it depended on the functionality of each tool, the clarity around what tools were used for which tasks, the synchronization between multiple tools, and the motivations to self-learn. Herein, team members had to sometimes navigate the lack of interoperability of multiple tools, especially when they relied on System 1 for Task A to progress to Task B on System 2, and System 1 lagged or glitched. Previous work has argued that “technology crowding” or diminishing marginal returns on additional tools for knowledge workers, can negatively impact productivity when there is information or communication overload (Karr-Wisniewski and Lu, 2010). Although we did not have enough occurrences to examine this as a separate theme, we noticed that prior familiarity with a tool or team members' own technological prowess appeared to influence its perceived effectiveness. Future research could examine how CTRTs might balance providing top-down standardized protocols and trainings while also giving members bottoms-up autonomy over the use these tools. Our findings further highlight the need for funding agencies and institutions to conduct adequate user research to better understand how mandated tools work in practice for scientists who regularly use them to conduct their large-scale projects. As one team observed, despite broad awareness of glitches in tool functionality at the consortium-level, the systems remained unchanged, with the CTRT expected to merely adjust. Parallel work in understanding the user effectiveness of data management systems in biomedical and clinical research has shed light on the facets of these systems that support effective data management (Ismail et al., 2020). Future work should similarly examine user experiences with information management tools (both homegrown by teams, and those that are commercially available) to better understand how CTRTs can be supported in their information endeavors.

Finally, our analysis underscored the role of team member compromises to achieve better team functionality. Specifically, amid the layers of information and tools, the daily work of team members depended on navigating both their own organizational approaches and the team's collective system. Several team members described themselves as being in a state of compromise, either striving to maintain a higher level of meticulousness or relinquishing organizational control for the sake of team functioning. For the most part, this compromise appeared to help the team move forward, as reflected in Theme 3, where individuals were able to see the bigger picture of the team's work. We note here that most teams had not engaged in explicit conversations or made decisions about these compromises, i.e. whether their current naming conventions or folder structures were working for everyone. A case study on a multidisciplinary multi-institution study revealed that team members constantly walked a “tightrope” between tasks, hierarchies, and tools (Lawrence, 2006), much as we observed. Importantly, the author argues that using more tools to streamline information would not solve these challenges; rather, conversations that clarify expectations and information use are key (Lawrence, 2006). Consistent with this, our findings suggest that to establish robust information practices, teams should take the time to discuss not only the broader tools they use for information management (almost all our teams had achieved this) but also the finer details of information organization. The few teams that had done so experienced greater transparency and collaboration, rather than frustration or conflict.

### 4.1 Limitations and future directions

Our findings are limited by the size and composition of the participating teams, as well as by who on the team chose to participate. While the number of team members interviewed ranged from three to seven, we do not have data on the full size of each team, i.e. all the members who might be considered as part of the project. Simulated modeling demonstrates that scientific teams typically contain of a “core” team (lead authors with their coauthors) and “extended” team (new members added proportional to the past productivity of the core members; Milojević, 2014). Similarly, our participants appeared to be the core members of their projects, but they tended to allude to other members whose level of involvement in the team is unclear to us. For instance, the full size of the one team that spanned across four labs could have included all the PIs, research staff, and their students (whom we did not interview), but not all of them would have been actively involved to the same extent in the project under study. Science teams tend to have permeable boundaries, and defining who is on the team is a recognized challenge in the team science literature (Cooke and Hilton, 2015). As such, distinguishing the collaborative information infrastructure of a smaller core team to those of an extended team reflects a broader issue of defining team membership for CTRTs. Moreover, although CTRTs are cross-disciplinary by definition, given the constraints to defining membership, we could not determine the extent to which the teams were uni- or cross-disciplinary as they varied in the types of collaborators they had across departments and institutions. Such distinctions could influence the degree of disciplinary differences that exist within teams which might contextualize their information expectations. Additionally, while our sample included different CTRT roles, it did not span the full range of personas that comprise a CTRT. For instance, we did not have members who were the formally appointed statisticians on their teams. Team science literature has highlighted the value of biostatisticians (and statisticians more generally) in academic health centers and CTRTs, especially their collaborative contributions to ensuring rigorous study design, providing analytical expertise, and enhancing publication success (Gonzales et al., 2020; Pomann et al., 2021; Slade et al., 2023; Spratt et al., 2017). It is conceivable that the information resources and tools needed by members of different roles could vary, and we are limited in our sample representation to uncover such needs.

This account is also limited by the relatively small number of teams. Our target sample was 15 teams, but recruitment proved challenging. We have documented our experience recruiting CTRTs in Venkatesh and Rolland (2025). Specifically, recruitment required multiple outreach strategies, but attaining buy-in from teams was hard. As this account shows, CTRT members already face challenges balancing multiple tasks, making it difficult to collectively commit to an additional activity. Moreover, as described earlier, some team members viewed information management as less of a priority when compared to their scientific goals, which may have reduced their incentive to participate. Investigating CTRTs' motivations to participate in studies about their collaborative work warrants further research. Nevertheless, studies like ours aim to uncover best practices to strengthen collaboration and team science. Importantly, this study is among the first to our awareness to examine information management with teams from across the country, representing whole teams rather than only individual members, thereby adding to our evidence-base for information management in CTRTs.

In forthcoming manuscripts from the IMPACT-CTR project, we will build on this foundation to continue along the Translational Team Science Hierarchy of Needs (Kelly et al., 2023) model to further examine the team processes in information management that impact collaborative success. This initial paper establishes evidence of the collaborative infrastructure that supports CTRTs, while subsequent papers will parse CTRTs' information strategies, workarounds, and lessons learned. For instance, we will examine how the Project Manager, who is involved in the regular upkeep of the Super Spreadsheet, perceives its functionality, compared to the PI, who refers to the sheet on an as-needed basis. Importantly, we will focus on people as a resource. In an exploratory top 200 words cloud on information types generated by NVivo, the word “people” appeared 84 times. Indeed, team members consistently relied on each other's expertise and knowledge. Given that our CTRTs include members with varying roles and hierarchies (Vanstone and Grierson, 2022), we will further delineate how team member knowledge shapes information practices. Taken together, this body of work seeks to contribute to translational team science by investigating intentional information management, such that our findings can be translated into practice by developers of team-based interventions and the teams themselves.

### 4.2 Conclusion

As science is increasingly conducted collaboratively, across disciplines and institutions, the information required to support collaboration, communication, and coordination becomes increasingly critical to the achievement of scientific objectives. Yet, as shown here and in our previous work, few teams recognize the impact their information management approaches have on their scientific and organizational work, and institutions are deploying tools that fail to facilitate and, sometimes, actively hinder the team's scientific progress. By describing the types of information, the complex constellation of tools, and the learning required for information management in CTRT, we can begin to focus attention on improving information management in the conduct of CTR. By shedding light on these dynamics of tools, resources, and people in CTRTs, we aim to make the *process* of doing science a little less fraught, so that the translational continuum can operate as efficiently and effectively as possible.

## Data Availability

The datasets presented in this article are not readily available because this is the first in a series of papers from the IMPACT-CTR study and the codebook is still under development. We will make the codebook available with subsequent manuscripts. Requests to access the datasets should be directed to Shruthi Venkatesh, vshruth@umich.edu.
