# Supplementary material for: The Super Spreadsheet: collaborative information infrastructure in translational teams
Source: Front Psychol. 2026 Jan 12;16:1724977. doi: 10.3389/fpsyg.2025.1724977 (PMC12833019; doi:10.3389/fpsyg.2025.1724977)
Supplement: Supplementary file 1 [file Data_Sheet_1.docx]

**Appendix**

**CTRT Team Roles**

For the purposes of analysis, we combined certain roles from the transcripts with those reported in the demographic survey to streamline categories (Table A1). The Lab Specialist, Research Investigator, and Research Scientist roles were grouped under “Research Specialist,” as their transcripts revealed similar responsibilities: senior scientists engaged in rigorous data management and analysis, along with oversight of other team members. The Data Manager was combined with the Postdoctoral Scholar; while these are distinct roles, in our sample, the postdocs primarily focused on data management and analysis. Three individuals who had selected Research Coordinator in the survey were grouped with Project Managers, as transcripts indicated they were in fact lead coordinators overseeing people, schedules, administrative tasks, and meeting agendas. Finally, student assistants (both graduate and undergraduate) were grouped together with Research Assistants under a broader Research Assistant category.

**Table A1:** Modified CTRT roles

| **Role as reported in survey** | **#** |  |  | **Modified role** | **#** |
| --- | --- | --- | --- | --- | --- |
| Faculty member | 19 |  |  | Faculty member | **19** |
|  |  |  |  | Staff | **16** |
| Data Manager | 1 |  |  | Postdoctoral Scholar | 3 |
| Postdoctoral Scholar | 2 |  |  |  |  |
| Lab Specialist | 1 |  |  | Research Specialist | 3 |
| Research Investigator | 1 |  |  |  |  |
| Research Scientist | 1 |  |  |  |  |
| Research Coordinator | 13 | **(-3)** |  | Research Coordinator | **10** |
| Program/Project Manager | 4 | **(+3)** |  | Program/Project Manager | **7** |
| Research Assistant | 3 |  |  | Research Assistant | **6** |
| Student Assistant | 3 |  |  |  |  |

**Interview Protocol**

Let's begin by talking a little bit about the team you are a part of/project.

1. Can you briefly explain your project? What is your team trying to achieve?
2. What does success look like for the team overall?

Now let’s chat a little bit about ways you interact with information including ways your team and you Seek, Use, Share, Store and Retrieve Information. Here, we use the term “information” to be any sort of digital object that is not project data, including, but not limited to, protocols, meeting minutes, communications, recruitment marketing materials, basically any of the sorts of information you use to do your work.

1. Let’s start with team meetings. How does your team record important decisions about the project or discussions at meetings?
   1. If you had an important decision to record for your team’s work, where would you put it?
   2. Who’s responsible for recording these decisions?
   3. Has your team explicitly discussed where to store the results of decisions. Are there written guidelines?
2. If you are writing a paper about the latest results, how would you go about gathering the information (not the data) you needed to describe how you did the work?
   1. How accurate and complete would that information be?
   2. If you were to write something collaboratively, how would you go about doing that?
3. How does your team record and share data? What types of information do you share? e.g. versions, metadata, data cleaning, data dictionary
   1. Who’s responsible for management of information around data?
4. Tell me about a time when you couldn’t find something you needed (For example, a document, an older version of the protocol, the decisions around patient recruitment)
5. What was the thing and what eventually happened?
6. What was the impact of being unable to find this piece of information?
7. Did you change your process after that?
8. Did you establish a process that others have subsequently used?
9. What did you and/or your team learn from this?
10. Tell me about a time when you didn’t know where to store or how to share something.
11. What was the thing and what eventually happened?
12. What was the impact of being unsure about where to store it or how to share it?
13. Did you change your process after that?
14. Did you establish a process that others have subsequently used?
15. What did you and/or your team learn from this?
16. On the flip side of that, tell me about a time when you found something or a process easy to navigate?
17. What was the thing and how was it stored/set up?
18. What about it did you find convenient?
19. How similar or different are your information management approaches (more broadly) to those of your team members?
20. How does this alignment or misalignment impact your work on this project?
21. What other kinds of information does your team deal with that we haven’t covered? Or other platforms you use that we haven’t discussed?
22. Your friend is starting a new lab or project. How would you advise them to set up their team’s approach to information?
23. Is there anything else you’d like to share about your team’s approach to information that we didn’t cover?
